# Leachable Poly(Trimethylene Carbonate)/CaCO_3_ Composites for Additive Manufacturing of Microporous Vascular Structures

**DOI:** 10.3390/ma13153435

**Published:** 2020-08-04

**Authors:** Zhengchao Guo, Dirk Grijpma, André Poot

**Affiliations:** Department of Biomaterials Science and Technology, University of Twente, 7522NB Enschede, The Netherlands; z.guo@utwente.nl (Z.G.); d.w.grijpma@utwente.nl (D.G.)

**Keywords:** poly(trimethylene carbonate), CaCO_3_, composite, additive manufacturing, stereolithography, microporous, vascular structures

## Abstract

The aim of this work was to fabricate microporous poly(trimethylene carbonate) (PTMC) vascular structures by stereolithography (SLA) for applications in tissue engineering and organ models. Leachable CaCO_3_ particles with an average size of 0.56 μm were used as porogens. Composites of photocrosslinkable PTMC and CaCO_3_ particles were cast on glass plates, crosslinked by ultraviolet light treatment and leached in watery HCl solutions. In order to obtain interconnected pore structures, the PTMC/CaCO_3_ composites had to contain at least 30 vol % CaCO_3_. Leached PTMC films had porosities ranging from 33% to 71% and a pore size of around 0.5 μm. The mechanical properties of the microporous PTMC films matched with those of natural blood vessels. Resins based on PTMC/CaCO_3_ composites with 45 vol % CaCO_3_ particles were formulated and successfully used to build vascular structures of various shapes and sizes by SLA. The intrinsic permeabilities of the microporous PTMC films and vascular structures were at least one order of magnitude higher than reported for the extracellular matrix, indicating no mass transfer limitations in the case of cell seeding.

## 1. Introduction

Fabrication of artificial vascular structures is not only needed for traditional tissue engineering applications, but for disease models on chip as well [1,2,3,4,5]. Large tissue engineering is regarded as a viable strategy for the regeneration of organs, which may provide a solution for the limited availability of donor organs for transplantation [6,7,8]. However, engineering of tissues remains a challenge, because the viability of seeded cells as well as in situ tissue formation are dependent on the presence of a vascular system [9,10,11]. Therefore, tissue engineering scaffolds as well as chip-based organ models have been developed both containing vascular structures, using 3D printing of sacrificial templates and 3D bioprinting [12,13,14,15,16,17].

Additive manufacturing (AM) allows for the preparation of designed tissue engineering scaffolds with optimal properties concerning porosity, pore interconnectivity, pore size and pore geometry. Of all AM techniques, stereolithography (SLA) is the most versatile and accurate method allowing structures to be built at a resolution of 10–150 μm [18,19]. Although for many applications pores sizes in this range or somewhat larger are suitable for cell seeding, the presence of (sub)micron-sized pores in the scaffold struts is advantageous in view of prolonged nutritional supply throughout the scaffold after implantation. Likewise, the walls of an artificial vascular network need to be microporous for the delivery of nutrients to cells and removal of waste products. Current commercial SLA machines, however, are not able to build pores in the (sub)micron range.

Porosity in tissue engineering scaffolds can be efficiently created by means of particle leaching [20]. NaCl and sugar particles are generally used for relatively large pores, ranging from tens to hundreds of micrometers, whereas micropores have been formed by leaching 2 μm ZnO crystals or 5–15 μm NaF particles [21]. We have recently shown that scaffolds for bone regeneration can be built by SLA using a polymer/nanohydroxyapatite composite [22]. Likewise, it should be possible to build vascular structures by SLA using a polymer/leachable particle composite. In this study, we used CaCO_3_ as a porogen, because of the uniform size of the particles which show little tendency to aggregate and can be easily leached.

Synthetic polymers are widely used to build structures by SLA for biomedical applications [23,24]. In previous work, we have used flexible poly(trimethylene carbonate) (PTMC) to prepare tubular scaffolds for vascular tissue engineering by dipcoating or molding [25,26]. PTMC is an amorphous rubber-like polymer that degrades by surface erosion in vivo without the formation of acidic degradation products [27,28,29]. Because of these characteristics, which are lacking in other polymers like poly(lactic acid) and poly(ε-caprolactone), PTMC is a very suitable material for vascular tissue engineering. The mechanical properties and degradation rate of PTMC networks can be tuned by varying the crosslink density as well as by copolymerization with, e.g., poly(lactic acid) and poly(ε-caprolactone) [28,29,30]. In a previous study, we have built a microvascular network from PTMC by SLA [31]. The capillaries had an inner diameter of approximately 200 μm and a wall thickness of 150 μm. The walls of the channels were nonporous, which would hamper the formation of tissue around the capillaries. Therefore, in the present study, we aimed to fabricate microporous PTMC vascular structures by SLA and subsequent particle leaching.

## 2. Materials and Methods

### 2.1. Materials

Trimethylene carbonate (TMC) monomer was kindly provided by Huizhou Foryou Medical Devices, Huizhou, China. 1,1,1-Tris(hydroxymethyl)propane, 2-Hydroxy-4′-(2-hydroxyethoxy)- 2-methyl-propiophenone (Irgacure 2959), Tin(II)-2-ethylhexanoate (Stannous octoate, Sn(Oct)_2_), hydroquinone, methacrylic anhydride and triethylamine were purchased from Sigma Aldrich, Zwijndrecht, The Netherlands. Propylene carbonate was ordered from Merck Millipore, Darmstadt, Germany. Ethyl-(2,4,6-trimethyl-benzoyl)-phenylphosphinate (Omnirad TPO-L) was obtained from IGM Resins, Waalwijk, The Netherlands. Orasol Orange G dye was ordered from CIBA Specialty Chemicals, Basel, Switzerland. Hydrochloric acid (37% (*w*/*w*) in water), analytical grade chloroform, dichloromethane (DCM), ethanol, methanol and acetone were purchased from VWR Chemicals, Darmstadt, Germany. CaCO_3_ was ordered from Alfa Aesar, Tewksbury, MA, USA.

### 2.2. Synthesis and Functionalization of Three-Armed PTMC

Three-armed PTMC was synthesized by ring-opening polymerization of TMC in a three-neck flask under argon atmosphere at 130 ℃ for 3 days [22]. 1,1,1-Tris(hydroxymethyl)propane and Sn(Oct)_2_ were used as initiator and catalyst, respectively. As resins for printing contained propylene carbonate diluent, three-armed PTMC was used to increase the possibility of crosslinking. The obtained PTMC was dissolved in DCM, and hydroquinone, triethylamine and methacrylic anhydride (MA) were added [22]. This was reacted at room temperature (RT) under argon protection for 5 days in the dark. PTMC-MA was obtained by precipitation in cold ethanol and drying in a vacuum oven at RT in the dark. The molecular weight (M_n_) and degree of functionalization of PTMC-MA were determined by ^1^H-NMR spectroscopy using an Ascend 400/Avance III 400 MHz NMR spectrometer (Bruker, Billerica, MA, USA).

### 2.3. Preparation and Characterization of PTMC-MA/CaCO_3_ Films

PTMC-MA and TPO-L were dissolved in chloroform (1 g PTMC-MA/3 mL chloroform, 5 wt % TPO-L relative to PTMC-MA). Various amounts of CaCO_3_ particles were dispersed in chloroform by sonication for 20 min. PTMC-MA/TPO-L solutions and CaCO_3_ dispersions were fully mixed and cast on glass plates using a casting knife. Chloroform was slowly evaporated overnight in the dark, after which the films were heated to 60 ℃ for 1 h to fully evaporate the remaining chloroform. The PTMC/CaCO_3_ composite films, containing 30–60 vol % CaCO_3_ particles in the polymer matrix, were photocrosslinked for 30 min in an ultraviolet (UV) light box at 365 nm wavelength and 8 mW/cm^2^ light intensity. To remove the sol fraction, the films were extracted for 3 days in chloroform, which was refreshed once per day. Finally, the composite films were immersed in ethanol and dried in a vacuum oven at 40 ℃ until constant weight.

The gel content of the photocrosslinked PTMC/CaCO_3_ composite films was determined by extraction of the sol fraction in chloroform as described above. The following equation was used,

(1) $$\mathrm{Gel}\;\mathrm{content}=\frac{m_{\mathrm{dry}}}{m_{\mathrm{initial}}}\times 100\%$$

in which m_dry_ is the mass of a PTMC/CaCO_3_ composite film after extraction and drying and m_initial_ is the mass of a photocrosslinked composite film before extraction.

### 2.4. Leaching of CaCO_3_ Particles and Characterization of Microporous PTMC Films

Photocrosslinked and extracted PTMC/CaCO_3_ composite films were immersed for 4 days in 3.7% (*w*/*w*) HCl solution in water, which was refreshed once per day. This yielded microporous PTMC films, which were finally soaked in distilled water.

The porosity of the photocrosslinked microporous PTMC films was determined gravimetrically according to the following equation:

(2) $$\mathrm{Porosity}=\left[1-\frac{m_{\mathrm{dry}}}{V\;\times \rho_{\mathrm{PTMC}}}\right]\times 100\%$$

in which ρ_PTMC_ = 1.31 g/cm^3^, m_dry_ is the dry weight of a microporous PTMC film and V the film’s bulk volume in either dry or hydrated state, yielding the porosity in dry or hydrated state, respectively.

### 2.5. Water Flux

Circular samples with a diameter of 26 mm were punched from hydrated microporous PTMC films. The samples were fixed in an Amicon cell 8003 (Merck Millipore, Darmstadt, Germany) with a filtration area of 0.9 cm^2^. MilliQ water was introduced onto the membranes at a pressure of 0.13, 0.23 or 0.33 bar. Upon reaching a stable water flow through the films, the permeating water mass was measured every 10 s for a minimum of 20 min.

### 2.6. Mechanical Properties

Samples with a length of 60 mm and a width of 5 mm were punched from the microporous PTMC films. The tensile properties of the films were determined in both hydrated and dry state using a Zwick Z020 tensile tester (ZwickRoell, Ulm, Germany). The initial grip to grip separation was 30 mm and a pulling rate of 50 mm/min was applied. The stiffness of the samples was determined from the slope of the stress–strain curve between 3% and 6% of strain.

### 2.7. Additive Manufacturing of Microvascular Structures Using PTMC/CaCO_3_ Resin

A dispersion of calculated amounts of CaCO_3_ and PTMC-MA in chloroform was homogenized and precipitated in cold ethanol to yield a composite that was dried in a vacuum oven at 40 ℃ to constant weight. Resins were prepared by homogenizing the composite in propylene carbonate and adding TPO-L photoinitiator and Orasol Orange G dye. The resin formulation is shown in Table 1. Structures were designed using Rhino 3D design software (Rhino 6, McNeel Europe, Barcelona, Spain). PTMC structures containing 45 vol % CaCO_3_ were printed using an Ember digital light processing stereolithograph (Autodesk, San Rafael, CA, USA) at a pixel resolution of 50 × 50 μm and a step height of 50 μm. Layers were sequentially photocrosslinked by exposure for 11 s to light with a wavelength of 405 nm and an intensity of 20 mW/cm^2^. Built structures were extracted for 4 days in chloroform/acetone (1:1 *v*/*v*) solution, which was refreshed once per day. Subsequently, the structures were dried and immersed for 1 day in chloroform containing 1% (*w*/*v*) Irgacure 2959, dried and postcured for 4 h in a UV cabinet at 254 nm and 10 mW/cm^2^. The CaCO_3_ particles were leached for 3 days in 3.7% (*w*/*w*) HCl solution in water, which was finally replaced by distilled water.

### 2.8. Scanning Electron Microscopy (SEM) and Thermogravimetric Analysis (TGA)

Samples were sputtered with gold using a Sputter Coater 108 Auto (Cressington, Watford, UK) set at 40 mA for 60 s. The average size of the CaCO_3_ particles was determined by measuring the size of 500 particles by SEM (JSM-IT100, JEOL, Tokyo, Japan). Surfaces and cross-sections of PTMC/CaCO_3_ composite films and printed microvascular structures, both before and after leaching of the CaCO_3_ particles, were also observed by SEM. CaCO_3_ content of the printed PTMC/CaCO_3_ microvascular structures was determined by TGA. The measurements were carried out using a temperature range of 50–550 ℃ at a heating rate of 20 ℃/min and a nitrogen flow of 20 mL/min (PerkinElmer, Pyris 1, Waltham, MA, USA).

## 3. Results and Discussion

### 3.1. Characterization of PTMC-MA, CaCO_3_ Particles and PTMC-MA/CaCO_3_ Composite Films

The synthesized PTMC-MA had a molecular weight (M_n_) of 4500 g/mol and degree of functionalization with methacrylate groups of 97%.

**Figure 1:**
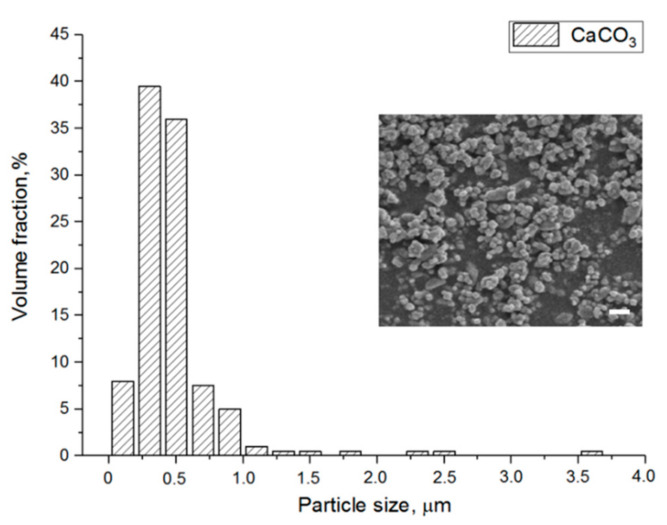
CaCO_3_ particle size distribution and SEM image of the particles. Scale bar 1 μm.

As shown in Figure 1, the CaCO_3_ particle size distribution ranged from 0.1 to 3.6 μm. The average particle size was 0.56 ± 0.32 μm, which indicated the potential of the CaCO_3_ particles as a leachable component given the intended SLA layer thickness of 50 μm.

To be able to leach all CaCO_3_ particles, the porous structure formed upon leaching should have interconnected pores. Therefore, a percolation threshold study was carried out, by preparing PTMC/CaCO_3_ composite films with 30, 40, 50 or 60 vol % CaCO_3_ particles in the polymer matrix. As shown in Table 2, the composite films had a gel content of at least 94.9%, which increased with a decreasing amount of CaCO_3_ particles. Pure PTMC films without CaCO_3_ had the highest gel content of 98.1%. Thus, although the presence of the particles slightly decreased UV crosslinking efficiency, the high gel contents indicated the formation of stable PTMC/CaCO_3_ composite films.

**Figure 2:**
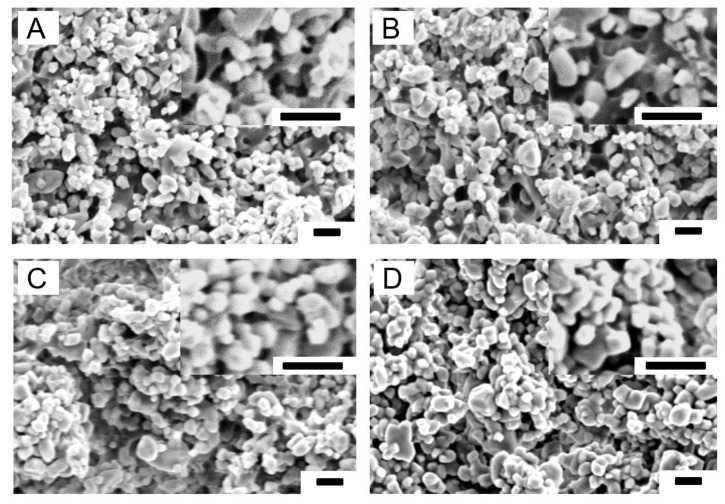
SEM pictures of cross-sections of PTMC/CaCO_3_ composite films. (**A**) PTMC/30; (**B**) PTMC/40; (**C**) PTMC/50; (**D**) PTMC/60. Scale bars 1 μm.

Figure 2 shows SEM images of cross-sections of the PTMC/CaCO_3_ composite films. With increasing CaCO_3_ loading more particles were observed, which were homogeneously distributed.

### 3.2. Characterization of Leached PTMC-MA/CaCO_3_ Composite Films

**Figure 3:**
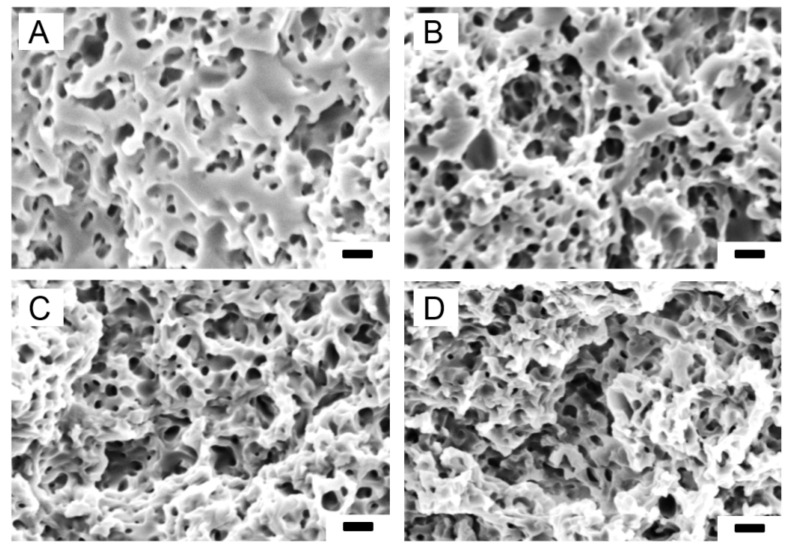
SEM pictures of cross-sections of leached PTMC/CaCO_3_ composite films. (**A**) PTMC/30; (**B**) PTMC/40; (**C**) PTMC/50; (**D**) PTMC/60. Scale bar 1 μm.

**Figure 4:**
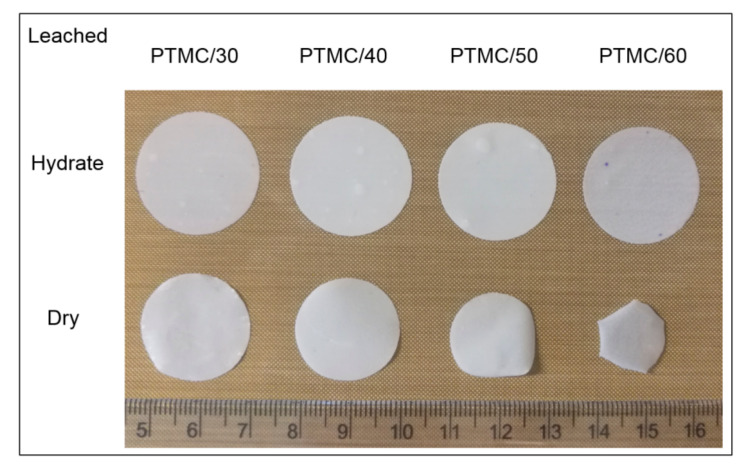
Macroscopic images of leached PTMC/CaCO_3_ composite films in hydrated and dry state.

All PTMC-MA/CaCO3 composite films were fully leachable in a 3.7% (*w*/*w*) HCl solution, see Figure 3. With increasing CaCO_3_ content more pores were observed, which were homogeneously distributed. The average pore sizes, determined from the SEM pictures, ranged from 0.45 to 0.50 μm (Table 3). This is somewhat smaller than the average size of the CaCO_3_ particles (0.56 μm). It should be noted that the pore sizes were determined in the dry state, which resulted in shrinkage of the leached films. This led to lower porosities of the films in the dry state as compared to the hydrated state, see Table 3. Moreover, the differences in porosities between dry and hydrated state increased with increasing CaCO_3_ content of the composite films. Thus, shrinkage of the dry films was higher at higher porosities, which is illustrated in Figure 4. Most probably, shrinkage of the leached films in the dry state was caused by the presence of pores, resulting in a relatively unstable structure, in combination with the low Tg of PTMC of around −20 ℃. In the hydrated state, the pores were filled with water which stabilized the structure. The thickness of the leached films in the hydrated state was around 120 μm.

### 3.3. Water Permeability of the Microporous PTMC Films

**Figure 5:**
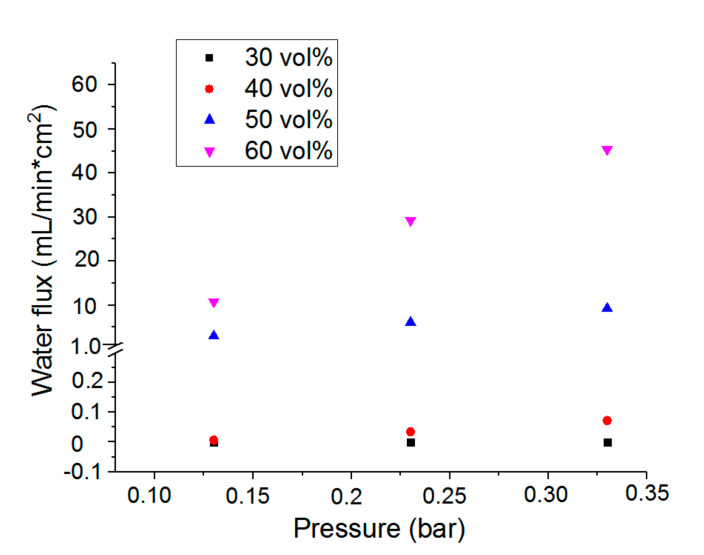
Water flux through the microporous PTMC films, vol % refers to the amount of CaCO_3_ particles used during preparation of the films.

For the delivery of nutrients to cells and removal of waste products, tissue engineering scaffolds should not only be porous but also permeable to watery solutions. This was tested for the microporous PTMC films by water flux measurements, see Figure 5. Except for the leached composite films with 30 vol % CaCO_3_, all other PTMC films showed a water flux through the microporous structures at pressures up to 0.33 bar. Water flux increased with increasing porosity of the films. Although the PTMC/30 composite films could be fully leached, indicating interconnected pores, a pressure of 0.33 bar in conjunction with the relatively low porosity of 33.2% was apparently not high enough to induce a flow of water through the films.

Water pressure of 0.16 bar is a physiological pressure corresponding to 120 mm Hg. The extrapolated water flux at 0.16 bar was used to calculate the intrinsic permeability of the PTMC/40, PTMC/50 and PTMC/60 microporous films according to Darcy’s formula [32]. The corresponding values were 3.13 × 10^−17^, 6.25 × 10^−15^ and 1.88 × 10^−14^ m^2^, respectively. This is at least 10-fold higher than the intrinsic permeability of 1.32 × 10^−18^ m^2^ reported for the extracellular matrix [33], indicating that these microporous structures would not hamper the delivery of nutrients to cells and removal of waste products.

### 3.4. Mechanical Properties of the Microporous PTMC Films

The mechanical properties of PTMC and microporous PTMC films are shown in Table 4. In the hydrated state, both stiffness (E_mod_) and maximum strength (F_max_) of the microporous films decreased with increasing porosity. Compared to dense PTMC films, the microporous films had lower E_mod_ and F_max_ and higher elongation at break due to the presence of pores. The same was observed for films in the dry state, albeit that E_mod_ and F_max_ decreased much less with increasing porosity due to the shrinkage of the microporous films upon drying.

The leached PTMC/50 films in the hydrated state had similar E_mod_, F_max_ and elongation at break as native blood vessels [25], indicating the suitability of these structures for cardiovascular applications.

### 3.5. Structure Design, Resin Formulation and SLA

**Figure 6:**
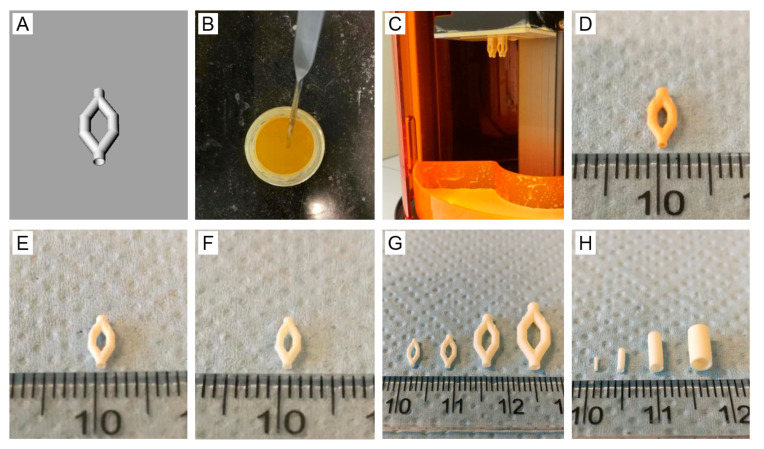
(**A**) design of branched vascular structure; (**B**) PTMC/CaCO_3_ composite resin; (**C**) built structures attached to printing head after SLA; (**D**) vascular structure before extraction; (**E**) vascular structure after extraction; (**F**) vascular structure after extraction, postcuring and leaching (hydrated); (**G**) branched vascular structures of different sizes (extracted, postcured and leached, hydrated); (**H**) vascular tubes of different sizes (extracted, postcured and leached, hydrated).

A branched vascular structure was designed as shown in Figure 6A. The resin for SLA-based printing contained PTMC, CaCO_3_, photoinitiator TPO-L and Orasol Orange G dye in propylene carbonate diluent. The composition of the resin is shown in Table 1 and a macroscopic image of the resin in Figure 6B. Based on the above percolation threshold study, 45 vol % CaCO_3_ relative to the PTMC matrix was chosen as a leachable component for the creation of a microporous structure. The Orasol Orange G dye content was optimized to ensure a proper curing depth as previously described [34]. Branched vascular structures were successfully built and extracted, see Figure 6D,E, respectively. As complete leaching of these structures was not possible due to pore collapse, a postcuring step was implemented after extraction. After printing in various shapes and sizes, postcured structures could be fully leached as shown in Figure 6F–H.

### 3.6. Characterization of the Branched Vascular PTMC Structures

**Figure 7:**
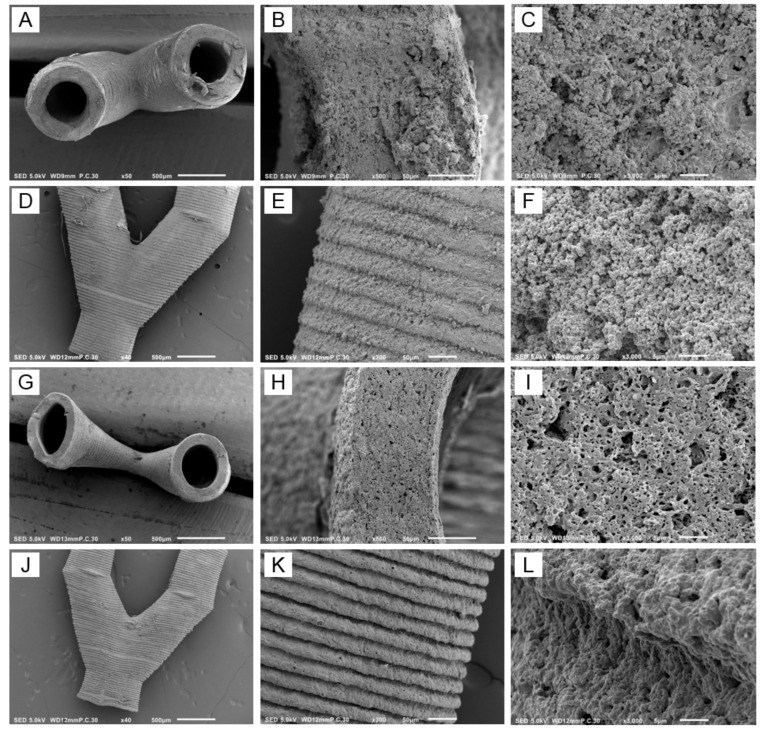
SEM images of (**A**–**F**) nonleached and (**G**–**L**) leached SLA-built vascular structures after extraction and postcuring. (**A**–**C**) and (**G**–**I**) show cross-sections, whereas (**D**–**F**) and (**J**–**L**) show the outer surfaces of the channels. Scale bar 500 μm (**A**,**D**,**G**,**J**), scale bar 50 μm (**B**,**E**,**H**,**K**), scale bar 5 μm (**C**,**F**,**I**,**L**).

SEM images of the vascular structures shown in Figure 6E,F are presented in Figure 7. Cross-sections showed branched open tubular structures, both in the case of nonleached and leached samples (Figure 7A,G, respectively). The outer surfaces clearly showed that the structures were built layer by layer, see Figure 7D,E,J,K. Cross-sections of nonleached samples showed CaCO_3_ particles, which were replaced by pores after leaching (Figure 7C,F,I,L, respectively).

As shown in Table 5, the leached vascular structures in the hydrated state had an inner diameter of 482 μm and a wall thickness of 146 μm. Both values were lower in the dry state, caused by shrinkage of the structures similar to observed for the films. Taking shrinkage into account, pore sizes in the dry state around 0.40 μm were in agreement with a mean particle size of 0.56 μm. On SEM pictures (Figure 7I,L), some larger pores were visible, probably formed by particle agglomerates. The CaCO_3_ content determined by TGA of the nonleached vascular structures was 50.2 vol % (Table 5), which is higher than the theoretical value of 45 vol %. This can be explained by the extraction of noncrosslinked PTMC before postcuring. This was also observed for PTMC/nanohydroxyapatite composite scaffolds fabricated by SLA [22]. The porosity of the leached vascular structures in the hydrated state was 59%, which is in agreement with the CaCO_3_ particle content. Again because of shrinkage, the porosity of the structures in the dry state was lower (35%). Based on the water flux through the walls of the vascular structures, as shown in Table 5, an intrinsic permeability of 0.61 × 10^−16^ m^2^ was calculated. This is 50-fold higher than reported for the extracellular matrix, see discussion above for the films, indicating that the branched vascular structures will be highly permeable to nutrients and cellular waste products.

As our previous microvascular PTMC network built by SLA facilitated the adhesion and proliferation of human umbilical vein endothelial cells [31], a good biocompatibility of the vascular structures printed in the present study is expected as well. This is supported by other studies, in which PTMC scaffolds built by SLA were shown to be biocompatible with human mesenchymal stem cells and annulus fibrosus cells [22,35,36]. Both in vitro and in vivo, photocrosslinked PTMC networks degrade by surface erosion. Degradation rate increases with increasing macromer molecular weight, i.e., decreasing crosslink density [28]. This also holds for networks prepared from linear PTMC crosslinked by γ-irradiation [37,38].

In previous work, we have prepared porous tubular scaffolds for vascular tissue engineering by sequential dipcoating and salt leaching [25]. Using this technique, it is not possible to fabricate branched structures. A strategy to implement branched vascular structures in scaffolds for tissue engineering or organ models is the use of sacrificial templates, e.g., of poly(vinyl alcohol) [16] or carbohydrate [17,39]. The template is immersed in a hydrogel matrix that is crosslinked, after which the template is leached. A drawback of this approach is the lack of a barrier between, e.g., endothelial cells seeded in the channels and other cells present in the surrounding matrix. Therefore, carbohydrate sacrificial templates were coated with thin layers of synthetic polymers such as poly(ε-caprolactone) [17] or poly(lactic-co-glycolic acid) [39]. The layers had a thickness of 10–50 μm and were made porous by phase separation or inclusion of leachable NaCl particles, respectively. Although interesting, the polymer coatings had relatively low tensile strengths around 85 kPa [17] and large pore sizes up to 50 μm [39].

Microporous vascular structures built by SLA have not been described in literature before. Composites of commercial resins and leachable NaCl particles were used to print cubes, pyramids and macroporous scaffolds [40], but not branched vascular structures. Moreover, the smallest particle sizes ranged from 75 to 180 μm, resulting in large pores [40]. Printing of branched porous vascular structures by SLA using a cytocompatible polyacrylate has been reported, but the designed pores had a diameter of 100 μm which could not be covered by endothelial cells [41]. This problem will not be encountered with the microporous vascular structures presented in the present paper.

## 4. Conclusions

Leachable CaCO_3_ particles with an average size of 0.56 μm were found to be suitable as porogens for the preparation of microporous PTMC films and vascular structures by casting and SLA, respectively. In order to obtain interconnected pore structures, the PTMC/CaCO_3_ composites had to contain at least 30 vol % CaCO_3_. The mechanical properties of the microporous PTMC films matched with those of natural blood vessels. The intrinsic permeabilities of the microporous PTMC films and vascular structures were at least one order of magnitude higher than reported for the extracellular matrix, indicating no mass transfer limitations in the case of cell seeding.

## Figures and Tables

**Table 1:** Resin formulation for stereolithography.

Component	Weight (g)	Content (%)
PTMC-MA	46.5	22.1
CaCO_3_	78.7	37.4
Propylene carbonate	85.0	40.5
TPO-L	2.3	5 *
Orasol Orange G	0.07	0.15 *

* relative to the mass of PTMC-MA.

**Table 2:** Gel contents of poly(trimethylene carbonate) (PTMC) and PTMC/CaCO_3_ composite films.

Sample Code	CaCO_3_ Loading, vol %	Gel Content, %
PTMC	0	98.1 ± 0.3
PTMC/30	30	97.2 ± 1.1
PTMC/40	40	97.4 ± 0.7
PTMC/50	50	96.6 ± 0.6
PTMC/60	60	94.9 ± 2.1

For all measurements, N = 4.

**Table 3:** Porosity and pore size of leached PTMC/CaCO_3_ composite films.

Sample Code	Porosity, % Hydrated	Porosity, % Dry	Pore Size, μm
PTMC/30	33.2 ± 1.9	31.4 ± 2.2	0.49 ± 0.27
PTMC/40	43.1 ± 2.4	41.9 ± 1.4	0.50 ± 0.21
PTMC/50	57.3 ± 3.7	52.1 ± 4.3	0.45 ± 0.36
PTMC/60	71.7 ± 5.1	51.3 ± 3.9	0.46 ± 0.29

For all measurements, N = 4, except for pore size N = 75.

**Table 4:** Mechanical properties of PTMC and microporous PTMC films in hydrated and dry conditions.

	Hydrated			Dry		
Sample Code	E_mod_, MPa	F_max_, MPa	Elongation at Break, %	E_mod_, MPa	F_max_, MPa	Elongation at Break, %
PTMC	8.16 ± 0.43	4.81 ± 0.89	67.1 ± 10.9	9.07 ± 0.34	6.57 ± 0.14	83.6 ± 4.2
PTMC/30	3.52 ± 0.16	3.28 ± 0.72	89.6 ± 12.5	7.76 ± 0.15	5.76 ± 1.21	121.7 ± 12.1
PTMC/40	2.72 ± 0.14	2.79 ± 0.52	109.4 ± 11.7	6.95 ± 0.17	4.58 ± 1.79	131.5 ± 14.2
PTMC/50	1.13 ± 0.05	1.48 ± 0.21	103.5 ± 8.1	5.82 ± 0.18	2.70 ± 0.54	143.3 ± 24.1
PTMC/60	0.30 ± 0.06	0.54 ± 0.04	104.1 ± 10.2	6.13 ± 0.16	2.16 ± 0.11	86.8 ± 19.9

For all measurements, N = 4.

**Table 5:** Parameters of SLA-printed vascular structures.

	Non-Leached	Leached, Hydrated	Leached, Dry
Inner diameter, μm	480.2 ± 10.9	482 ± 10.2	416.3 ± 7.5
Wall thickness, μm	162.5 ± 4.3	146.0 ± 6.1	90.1 ± 3.8
Pore size, μm	-	-	0.40 ± 0.27
CaCO_3_ content, vol %	50.2 ± 2.9	-	-
Porosity, %	-	59 ± 3	35 ± 4
Water flux at 0.16 bar, mL/min·cm2	-	0.09 ± 0.02	-

For all measurements, N = 4, except for pore size N = 75.
